# Amputation at the Wrist-Joint, the Patient Being under the Influence of Chloroform

**Published:** 1848-11

**Authors:** Bela Colegrove

**Affiliations:** Sardinia, Erie Co.


*Amputation at the Wrist-Joint, the Patient being Under the Influence of Chloroform.* By Bela Colegrove, M. D., of Sardinia, Erie Co.

We introduce here, the following letter, which was received after the original contributions for this number were in print. Editor.

*To the Editor of the Buffalo Medical Journal:*

I accompanied my father to Boston in this county, Oct. 21, for the purpose of witnessing the removal of a mutilated hand. The patient, aged 20, was of muscular frame and vigorous constitution. Late in the afternoon of the previous day, while attempting to adjust the machinery of a saw-mill, his left hand coming in contact with the saw, was nearly divided at a single stroke. Upon examination, the meta-carpal bones were found to be completely cut through, and the soft parts of the hand extensively lacerated. The necessity of an operation becoming at once evident, a few drops of chloroform were applied to a sponge, and directed to be inhaled. A few inspirations sufficed to induce partial insensibility, and the knife was entered a little below the scaphoides, and carried steadily toward the point of connection between the radius and carpus. By a similar movement from the opposite side, the removal having been effected, the arteries were as usual secured, and the flaps handsomely adjusted. During the operation, the patient manifested little uneasiness, having only a vague and suppressed sense of suffering, which was shown by a low moaning. The influence of the anæsthetic agent, was conspicuously and happily exemplified. No untoward effect followed its administration, and it was reasonable to expect no accident to the stump. From an observation of its effects in numerous instances, it seems impossible to apprehend any serious casualty, when proper caution is exercised. It is believed that in almost every instance of fatal or unhappy issue, the indispensible condition to its safe administration was neglected to be observed. The spirit should not be *concentrated.* As it is incapable of supporting respiration, it can by no means supplant the introduction of atmospheric air with impunity. As oxygen is essential to respiration, it would seem superfluous to suggest that it must be admitted to the lungs *and freely respired with the narcotizing vapor.* The induction of complete insensibility can hardly be regarded as hazardous with the precaution just intimated. That it is poisonous is inadmissible. That its specific impression is hostile to the proper decarbonization of the blood, is incompatible with careful observation. And it must be equally obvious that the quantity should be adapted to the constitution of the patient. The specific effect is observed to supervene much more readily in some cases than others. These observations are not intended to reflect on the discrimination of those who have encountered unexpected mischief from its employment, but it is thought their propriety must occur to the reflective mind.

As an expedient for alleviating pain, or overcoming the sensibility to the knife, the chloroform must be acknowledged alike admirable and effectual.

Yours truly,

C. Colegrove.

